# Public trust in the time of the Coronavirus Disease 2019 (COVID-19): the case of the DR Congo

**DOI:** 10.11604/pamj.supp.2020.35.2.22606

**Published:** 2020-04-09

**Authors:** Guy-Lucien Whembolua, Daudet Ilunga Tshiswaka

**Affiliations:** 1 Department of Africana Studies, University of Cincinnati, Cincinnati, USA; 2 Department of Public Health, University of West Florida, Florida, USA

**Keywords:** Congo, COVID-19, trust, PEN-3

## Abstract

Over the past half century, the Democratic Republic of the Congo (DRC), a low-income and post-conflict country, has experienced several Ebola Virus Disease outbreaks, with different fatality rates. The DRC is currently experiencing the Coronavirus Disease 2019 (COVID-19) pandemic. Using the PEN-3 cultural model, we assessed the socio-cultural factors affecting public trust in the government and its health agencies. Results of this analysis revealed the perceptions, enablers, and nurturers that impacted public trust in the government and its health agencies among the Congolese population. Future interventions designed to address the COVID-19 in the DRC should account for these socio-cultural factors.

## Essay

As the world collectively navigates how to face the Coronavirus Disease 2019 (COVID-19) pandemic, it is crucial to highlight how low-income and post-conflict countries such as the Democratic Republic of Congo (DRC) experiences COVID-19. More specifically, using the PEN-3 cultural model, we assessed the socio-cultural factors affecting public trust in the government and its agencies (i.e., the main regulator of public health in the country) prior to the announcement of the first reported COVID-19 case on March 10th 2020, one day before the World Health Organization declared the disease a pandemic [1]. The PEN-3 cultural model has been used as a guide to explore health issues by situating them within their socio-cultural contexts to inform policymakers or interventionists [2]. The theoretical model was developed by Airhihenbuwa (1989) to examine culture and its impact on health behaviors and beliefs through the lens of three main domains, including cultural identity, relationships and expectations, and cultural empowerment [3]. For instance, the use of the relationships and expectations domain has proven to be insightful during the Ebola virus disease crisis, revealing revealed the perceptions and enabling/nurturing factors that exacerbated to or prevented Ebola Virus Disease-related stigma [4]. The Relationships and Expectations domain considers elements that influence health behaviors and decisions such as perceptions, enablers, and nurturers that increased public mistrust in the government and its health agencies among the Congolese population.

According to the PEN-3 model, perception is defined as beliefs, knowledge or attitudes about COVID-19 and health agencies that impact public trust into the Congolese institutions [5]. Prior to the announcement of the first ever reported case, there were reports circulating through newspapers in the DRC that people of Black descent and/or those living in hot and humid climates could not be infected by COVID-19. These rumors were amplified by a Twitter post made by a preeminent member of parliament stating that “Coronovirus is a hoax!” [6]. Another rumor stated by a well-known evangelical pastor maintained that coronavirus was laboratory-made by multinational companies to control population on earth [7]. Enabling factors-another tenet of the PEN-3 model-refer to institutional support and assets at the structural level that foster or deter health-seeking practices. Several lingering factors existing at the institutional level contribute to mistrust towards any governmental information. In 2014, the DRC, through the government health initiative, built the second largest and well-equipped medical center in the capital city (Kinshasa). Initially, this modern medical center wanted to be affordable and efficient for the general population. Unfortunately, the hospital ended up performing poorly in terms of patient survival and safety [8]. Moreover, the high level of inefficiency and corruption present in the state apparatus (the country currently ranks at 168 out 180 country in the transparency international corruption index) is perceived as one of the largest hindrances towards a containment of COVID-19. [9]. This was illustrated by the statement “Anyone can easily enter the country without being tested in exchange for a payment” made during a presentation at the national assembly by one of the most senior virologists, a world-known Ebola researcher [10].

Following the PEN-3 cultural model, the influence of messages shared by local people that contribute mistrust of governmental agencies is classified as nurturers, which are the third tenet of the Relationships and Expectations domain. Interestingly, it is now well understood that outbreaks can create a complex collective experience shared by communities that nurture health-seeking practices [11]. According to the World Bank, the DRC has 9% Internet penetration, representing an extremely low proportion [12]. However, communication through cell phones is much more widespread [13]. The use of memes, videos, screenshots sent via cellular phones intensified governmental mistrust. In one of these videos, a music band sang a tune claiming that Coronavirus was created by Western countries [14]. Another video depicts young men appearing to be “Shegués”, (i.e., a Congolese term referring to a homeless child who lives on the street) stating that they will never be infected by Coronavirus [15]. A screenshot of Facebook post made by a famous Congolese journalist advised the inhalation of a well-known commercial topical Cough Suppressant in order to prevent any infection by the virus.

Consequently, despite limited official communication, confusion and mistrust gained ground in the general population following the announcement of the first DRC case of COVID-19. Indeed, the first reported COVID-19 case in the DRC involved a Congolese citizen residing in France who had come to visit relatives in DRC two days earlier. Unprepared to handle coronavirus cases-although local authorities have previously stated otherwise-the first case was forced into quarantine at a local hospital from which some patients and healthcare professionals ran away when they learned that a COVID-19 patient was admitted, creating an unprecedented chaotic situation [16]. Considering that the patient was asymptomatic and the lack of adequate communication from medical/health authorities, skepticism, suspicion, and mistrust increased within the general population. Ultimately, it took the DRC government eight days after the first reported case to finally announce a series of stringent measures to tackle this pandemic [16]. These measures included the closing of all universities, schools for four weeks, the interdiction of all gatherings of more than 20 individuals and the suspension of all flights from coronavirus-infected countries. At the date of this writing, DRC has now reported 45 COVID-19 cases, with three deaths due to disease complications [16]. In the near future, Congolese health leaders may have to build on the DRC past experience with the Ebola Virus Disease where local leaders and savoir-faire were used not only to contain the disease, but to also launch multi-media prevention campaign that addresses all the different inaccurate information currently spread in the population.

## Competing interests

The authors declare no competing interests.
